# Variations in facial conformation are associated with differences in nasal microbiota in healthy dogs

**DOI:** 10.1186/s12917-021-03055-w

**Published:** 2021-11-24

**Authors:** Emilie Vangrinsven, Aline Fastrès, Bernard Taminiau, Billen Frédéric, Georges Daube, Cécile Clercx

**Affiliations:** 1 Department of Clinical Sciences, Faculty of Veterinary Medicine, University of Liège, Quartier Vallée 2, Avenue de Cureghem 3, 4000 Liège, Belgium; 2 Department of Food Sciences - Microbiology, Faculty of Veterinary Medicine, University of Liège, Quartier Vallée 2, Avenue de Cureghem 3, 4000 Liège, Belgium

**Keywords:** Nasal cavity, Microbiota, Dogs, Breed, Facial conformation

## Abstract

**Background:**

Extrinsic and intrinsic factors have been shown to influence nasal microbiota (NM) in humans. Very few studies investigated the association between nasal microbiota and factors such as facial/body conformation, age, and environment in dogs. The objectives are to investigate variations in NM in healthy dogs with different facial and body conformations. A total of 46 dogs of different age, living environment and from 3 different breed groups were recruited: 22 meso−/dolichocephalic medium to large breed dogs, 12 brachycephalic dogs and 12 terrier breeds. The nasal bacterial microbiota was assessed through sequencing of 16S rRNA gene (V1-V3 regions) amplicons.

**Results:**

We showed major differences in the NM composition together with increased richness and α-diversity in brachycephalic dogs, compared to meso−/dolichocephalic medium to large dogs and dogs from terrier breeds.

**Conclusion:**

Healthy brachycephalic breeds and their unique facial conformation is associated with a distinct NM profile. Description of the NM in healthy dogs serves as a foundation for future researches assessing the changes associated with disease and the modulation of NM communities as a potential treatment.

## Background

Studies investigating the composition of the microbial communities of the nasal cavities in healthy individuals using culture-independent molecular methods have arisen over the last decade in humans [1–6] and more recently in companion animal veterinary medicine [7–11].

In healthy humans, both extrinsic and intrinsic factors have been reported to shape an individual’s upper airway microbiome. Extrinsic exposures reported to influence the nasal microbiota (NM) composition include delivery at birth mode [12] and feeding type [13] in infants, and exposure to tobacco smoke [14] and air pollution [15] in adults.

Intrinsic factors like host genetics [16] and associated host mucosal immunity [17] as well as age [18] also seem to modulate the NM. Moreover, in specific anatomic sites in the upper respiratory tract as well as within the nasal cavities, distinct micro-niches containing specialized bacterial communities have been described, demonstrating intra-patient spatial variation of the NM [2].

Studies in humans suggest that the NM is a key factor in maintaining respiratory health by affecting both resistance to pathogens and immunological responses [19–21]. While a greater mucosal diversity may play a role in limiting inflammation and protecting against infections [22–24], potential microbial species known as “keystone species” may also have a beneficial effect on the ecosystem’s balance, function and health [21, 25, 26].

In dogs, complex bacterial communities dominated by the Moraxellaceae family have been detected in the nasal cavities of healthy animals [7, 9, 10]. Spatial variations between nasal and oropharyngeal swabs [7, 9], temporal variations [9], changes associated with geographical location [9] as well as variations associated with older age and lower bodyweight [10] have been reported suggesting that extrinsic and intrinsic factors also influence upper airway bacterial composition in dogs.

Variable predispositions for chronic nasal disease are observed between different dog breeds. Indeed, chronic lymphoplasmacytic rhinitis (LPR) and sinonasal aspergillosis (SNA) are almost exclusively encountered in middle-aged, medium to large meso- or dolichocephalic canine breeds [27, 28]. In contrast, brachycephalic breeds, in which extreme breeding selection has progressively led to very distinct anatomical features of the nasal cavities [29, 30] are rarely affected by chronic inflammatory or infectious nasal diseases. Anatomical abnormalities described in the nasal cavities of brachycephalic dogs include marked global reduction of the size of the nasal cavities, stenosis of the nares and vestibulum, malformed rostral and caudal aberrant turbinates. Those abnormalities are associated with intranasal contact points, reduced lumen and increased collapsibility of the nasopharynx and missing paranasal sinuses. They cause abnormal breathing patterns, differences in airflow distribution and increased resistance to airflow [31–33]. Very few studies investigated the association between the NM and the facial/body conformation [10] or breed [9]. One study [10] compared the NM in dogs with different cephalic indexes (meso- and dolichocephalic) and failed to observe differences in nasal microbial composition. In the same study, dogs with a body weight of less than 10 kg had a significantly higher Shannon diversity index and species richness than dogs with a body weight over 10 kg. In another study [9], no differences in nasal bacterial communities were reported in dogs from different large dolichocephalic breeds.

We hypothesized that differences in facial anatomical conformation and consequently in breathing/airflow pattern might result in different nasal microbial composition. Therefore, the aim of this study was to describe and compare the composition of the NM in healthy dogs of various breeds, categorized into 3 groups according to their body and facial conformation.

## Results

### Study population

Forty-six client-owned dogs were recruited between November 2017 and July 2018. Groups DL (meso−/dolichocephalic dogs of medium to large breeds), B (brachycephalic breeds) and T (terrier breeds) contained 22, 12 and 12 dogs respectively.

### Breed, age, sex and bodyweight distribution

Group DL included mixed breed (*n* = 5), Belgian Shepherd (*n* = 4), Australian Shepherd (n = 4), Labrador (*n* = 2), Beauceron (*n* = 2), Border Collie (*n* = 2), Golden retriever (*n* = 1), Dalmatian (n = 1), and White Swiss Shepherd (n = 1). French Bulldog (*n* = 6), English Bulldog (*n* = 2), Pug (n = 2) and Cavalier King Charles Spaniel (n = 2) composed the group B. Group T was composed of 4 breeds: Jack Russel terrier (*n* = 7), Yorkshire terrier (*n* = 3), West Highland white terrier (*n* = 1) and Pinscher (n = 1). All dogs were healthy and from different households. The distribution of age, bodyweight and sex among groups is reported in Table 1. Dogs of group B were younger (more dogs in age class 1) compared to group T (more dogs in age class 2 and 3; *p* = 0.02). Median age for all dogs was 6 years with one 6 months old dog in group B and one 8 months old dog in group DL, one dog of 10.8 years in group T and two dogs of 10.4 and 11.3 years in group DL. The remaining dogs were between 1 and 10 years of age. As expected, all three groups had marked differences in bodyweight associated with their breed type. Gender distribution was comparable in the 3 groups.

**Table 1:** Distribution of age, bodyweight, sex, and living environment among the 3 breed groups of healthy dogs

	Group DL (*n* = 22)	Group B (*n* = 12)	Group T (n = 12)
Age mean (min-max)	5.8 (0.8–11.3)	3.9 (0.6–9.2)	6.8 (2.6–10.8)
Age classes n, %	class 1: 5, 23%	class 1: 5, 42%	class 1: 0, 0%
	class 2: 13, 59%	class 2: 6, 50%	class 2: 10, 83%
	class 3: 4, 18%	class 3: 1, 8%	class 3: 2, 17%
Bodyweight mean (min-max), kg	28 (14.8–41)	13.1 (6.8–21.4)	7.1 (2.9–10)
Female n, %	14, 64%	5, 42%	7, 58%
City n, %	7, 32%	4, 33%	3, 25%

Age class 1: < 3 years, class 2: 3–8 years and class 3: > 8 years

Group DL = mesocephalic or dolichocephalic dogs of medium to large breeds

Group B = brachycephalic dogs of small breeds

Group T = mesocephalic or dolichocephalic terrier dogs of small breeds

### Living environment and diet

Distribution of living environment was similar between groups (Table 1). Contact with tobacco smoke and food type were not taken into account because no dog lived in close contact with tobacco smoke (smokers were not smoking inside the house or in a place where the dog could be in regular contact with tobacco smoke) and because all dogs were fed with commercial dry food, at the exception of 4 of them (1 dog in group DL and 2 dogs in group T were eating a mix of commercial dry food and home-made diet; 1 dog in group B was eating a home-made diet). For these parameters, groups were too small to perform statistical analysis.

### Most common taxa

**Fig. 1:**
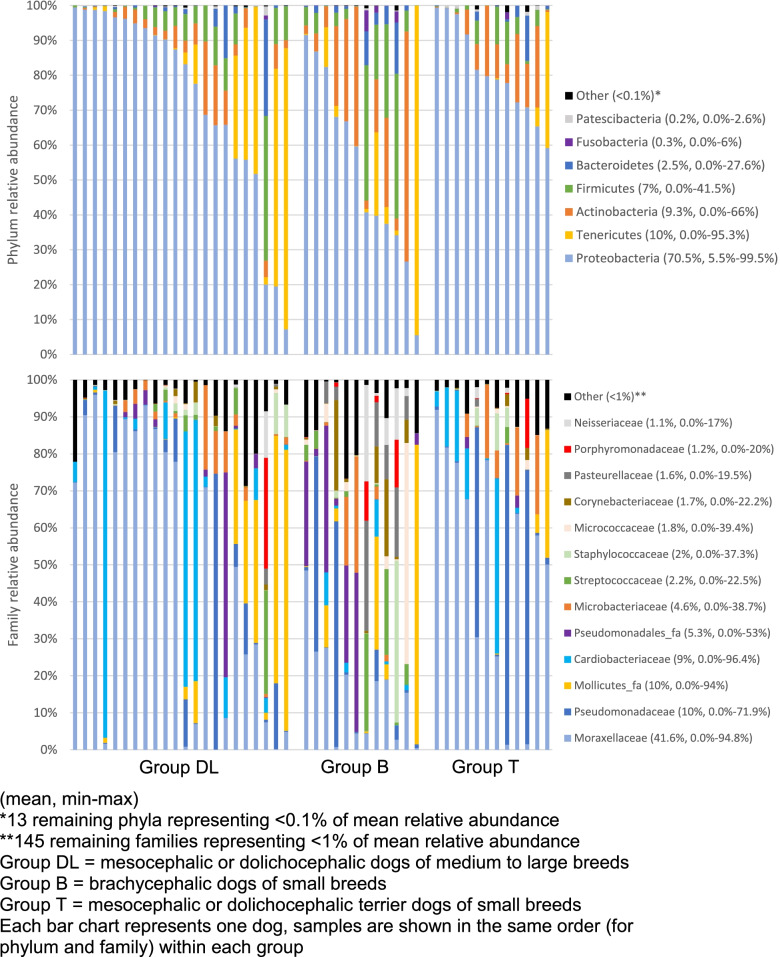
Composition of nasal microbiota at phylum and family level in healthy dogs from 3 different breed groups

Good’s coverage index was superior to 99% in all dogs with a median value of 99.81% (99.70–99.88%) indicating an equal and adequate sampling effort for all dogs. At the finest taxonomic resolution, a total of 755 operational taxonomic units (OTUs) were found throughout all samples. The most common taxa at phylum and family level in healthy dogs from different breed groups are illustrated in Fig. 1. A total of 20 phyla were identified in the samples of the nasal cavities of healthy dogs, the most abundant being Proteobacteria followed by Tenericutes, Actinobacteria, Firmicutes and Bacteroidetes. Other phyla such as Fusobacteria and Patescibacteria represented less than 1% of the mean relative abundance.

The most common taxon at the family level was Moraxellaceae representing 41.6% of the total taxa: this family was detected in all samples at percentages ranging from 0.04 to 94.8%. Pseudomonadaceae, unclassified family of the Mollicutes class, Cardiobacteriaceae, unclassified family of the Pseudomonadales order, Microbacteriaceae, Streptococcaceae and Staphylococcaceae constituted the major part of the other frequently detected families although some of them were present in large amounts in only a few animals. *Moraxella* was the predominant genus in the majority of samples. Together with the genera *Pseudomonas*, *Suttonella*, *Leucobacter*, and unclassified genera of the class Mollicutes and of the order Pseudomonadales they represented 80.1% of all taxa throughout all samples.

## Influence of age, sex, living environment and breed groups (DL, B and T) on the nasal microbiota

### Constrained ordination

Redundancy analysis (RDA) based on the table of values at species level showed that first breed group (Adjusted R^2^ 0.045 for group B versus group DL and T) and then to a lesser extent age classes (Adjusted R^2^ 0.006 for age class 1 versus age class 2 and 3) contributed to the variability of the microbiota (explaining together 12.9% of the variance).

### Richness, evenness and α-diversity

A similar RDA was performed based on richness (Chao1 index), evenness (Simpson index) and α-diversity (inverse Simpson index). Only breed groups contributed to the constraint (Adjusted R^2^ 0.074, explaining 11% of the variance for group B versus group DL and T).

**Fig. 2:**
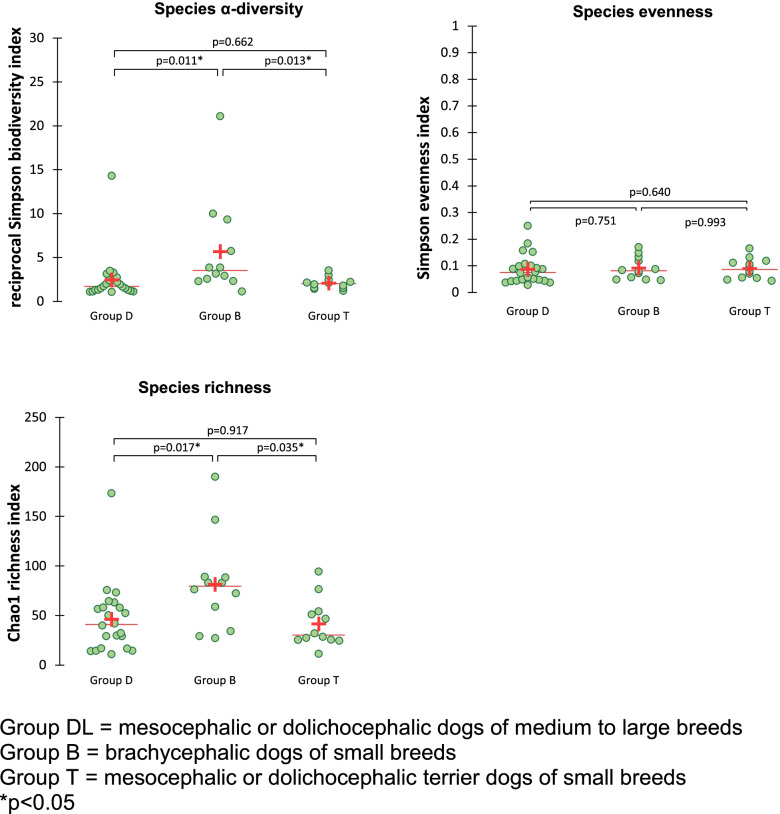
Richness, evenness and α-diversity in healthy dogs from 3 different breed groups. Group DL = mesocephalic or dolichocephalic dogs of medium to large breeds. Group B = brachycephalic dogs of small breeds. Group T = mesocephalic or dolichocephalic terrier dogs of small breeds. *p < 0.05

There were no differences in richness, α-diversity or evenness across the different age groups, sexes or living environments when these parameters were evaluated individually. Among the breed groups, Group B had a significantly higher species α-diversity and richness compared to group DL and T (Fig. 2). Evenness did not differ significantly between breed groups.

### Beta-diversity

**Fig. 3:**
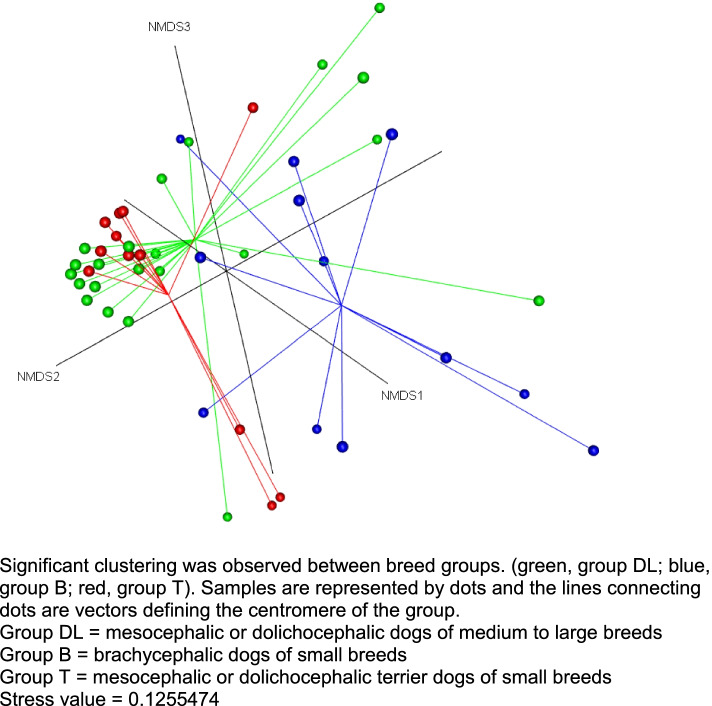
Non-metric multidimensional scaling (NMDS) ordination of nasal microbiota communities using Bray-Curtis in healthy dogs from 3 different breed groups. Significant clustering was observed between breed groups. (green, group DL; blue, group B; red, group T). Samples are represented by dots and the lines connecting dots are vectors defining the centromere of the group. Group DL = mesocephalic or dolichocephalic dogs of medium to large breeds. Group B = brachycephalic dogs of small breeds. Group T = mesocephalic or dolichocephalic terrier dogs of small breeds. Stress value = 0.1255474

When using an analysis of molecular variance (AMOVA) at species level, the β-diversity was not different between the age (*p* = 0.29), the sex (*p* = 0.63), or the living environment (*p* = 0.65) groups. The analysis of the molecular variance homogeneity (HOMOVA) at species level also did not reveal a significant difference between these groups (age, *p* = 0.86; sex, *p* = 0.49; living environment, *p* = 0.72). This was also suggested by the non-metric multidimensional scaling (NMDS) plot of microbial communities at species level based on a Bray-Curtis dissimilarity matrix which failed to reveal any clustering for these three exposures. In contrast, NMDS plot revealed a distinct separation between group B and the 2 other groups DL and T (Fig. 3). A significant difference in bacterial community composition was verified by an AMOVA analysis (*p* = 0.02) and pairwise comparison demonstrated that group B was different compared to groups DL and T (group B vs group DL *p* = 0.017; group B vs group T *p* = 0.008; group DL vs group T *p* = 0.511). The analysis of the molecular variance homogeneity (HOMOVA) did not reveal a significant difference between breed groups (*p* = 0.08).

## Bacterial composition in the nasal cavity of dogs from different breed groups

Based on the results of RDA, richness, evenness, α-diversity and β-diversity, breed group is the dominant variable influencing the nasal microbiota within the variables that were evaluated. Further investigation of bacterial composition was therefore performed by comparing the breed groups.

### Most common taxa

**Fig. 4:**
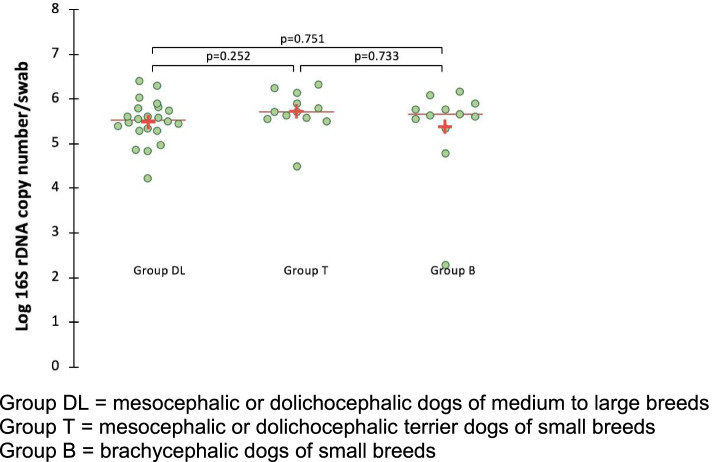
Total bacterial flora in healthy dogs from 3 different breed groups. Group DL = mesocephalic or dolichocephalic dogs of medium to large breeds. Group T = mesocephalic or dolichocephalic terrier dogs of small breeds. Group B = brachycephalic dogs of small breeds

The total bacterial flora quantified by 16S rRNA gene based qPCR did not significantly differ between groups of different breeds (Fig. 4).

Despite the fact that the community composition at the phylum level showed a dog-to-dog variation within each group (Fig. 1), the most abundant phylum throughout the 3 groups was Proteobacteria since it was the most predominant (more than 50%) in all dogs of group T, 6/12 dogs of group B and 19/22 dogs of group DL.

**Fig. 5:**
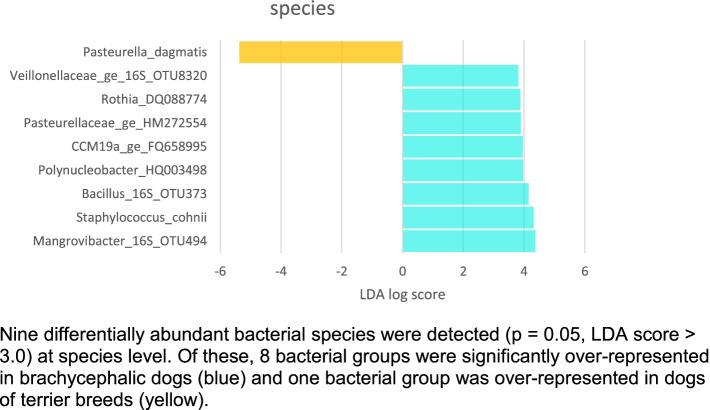
Linear discriminant analysis (LDA) effect size (LEfSe) at species level of Illumina sequencing datasets based on 16S rRNA gene sequences. Nine differentially abundant bacterial species were detected (*p* = 0.05, LDA score > 3.0) at species level. Of these, 8 bacterial groups were significantly over-represented in brachycephalic dogs (blue) and one bacterial group was over-represented in dogs of terrier breeds (yellow)

Linear discriminant analysis (LDA) effect size (LEfSe) scores indicated bacterial taxa that were mainly present in group B. The genera *Haemophilus* (family Pasteurellaceae), *Parvimonas* (family Clostridiales_Family_XI*)*, unclassified genus within the family Pasteurellaceae, *Fusobacterium* (family Fusobactericiaea), *Gemella* (family Bacillales_Family_XI), *Rothia* (family Micrococcaceae), *Conchiformibius* (family Neisseriaceae*)*, *Abiotrophia* (family Aerococcaceae) and *Streptococcus* (family Streptococcaceae) were significantly more represented in dogs from the group B. At species level 8 taxa were enriched in group B while only one taxon was associated with group T (Fig. 5).

### Differences in relative abundances

Mean relative abundances of taxa that significantly differ between groups can be found in Table 2. At the phylum level the proportion of Proteobacteria was significantly lower in group B, in comparison with DL and T, while the proportion of Actinobacteria, Firmicutes and other Proteobacteria was increased. In the phylum Proteobacteria, the genera *Moraxella* (family Moraxellaceae) and *Suttonella* (family Cardiobacteriaceae) were significantly lower in group B. Genera of other Proteobacteria that were more frequently observed in group B are an unclassified genus within the Pseudomonadales order as well as *Conchiformibus* (family Neisseriaceae) and an unclassified genus of the Pasteurellaceae family. Among the phylum Actinobacteria, the genera *Corynebacterium (*family *Corynebacteriaceae)* and *Rothia* (family Micrococcaceae) were more abundant in group B, although this was not significant. Among the phylum Firmicutes, *Streptococcus* (family *Streptococcaceae*), *Staphylococcus* (family Staphylococcaceae), *Abiotrophia* (family Aerococcaceae) and *Gemella* (family Bacillales_Family_XI) were more abundant (however not significantly) in group B.

**Table 2:** Bacterial groups at > 1% mean relative abundance among the 3 breed groups of healthy dogs at phylum, family and genus level

Taxon	Group DL			Group B			Group T		
Phylum	Mean rel. Freq. (%)	SD (%)	Tukey’s multiple comparisons test (p < 0.05)	Mean rel. Freq. (%)	SD (%)	Tukey’s multiple comparisons test (p < 0.05)	Mean rel. Freq. (%)	SD (%)	Tukey’s multiple comparisons test (p < 0.05)
Family									
*Genus*									
**Proteobacteria**	73.5	27.6	A***	53.3	25.6	**B**	81.1	12.8	A****
**Moraxellaceae**	48.9	38.1	A****	16.4	15.7	**B**	53.5	30.1	A****
*Moraxella*	48.9	38.1	A****	16.3	15.7	**B**	53.0	30.7	A****
**Pseudomonadaceae**	6.8	13.0	**A**	10.0	19.2	**AB**	16.0	27.1	B**
*Pseudomonas*	6.8	13.0	NS	10.0	19.2	NS	15.8	26.8	NS
**Pseudomonadales_fa**	3.1	10.0	A**	14.0	19.1	**B**	0.6	1.2	A***
*Pseudomonadales_ge*	3.1	10.0	A**	14.0	19.1	**B**	0.6	1.2	A**
**Cardiobacteriaceae**	12.8	26.7	A**	1.9	3.0	**B**	8.8	14.2	**AB**
*Suttonella*	12.8	26.7	A**	1.9	3.0	**B**	8.8	14.2	**AB**
**Pasteurellaceae**	0.2	0.6	NS	5.8	6.3	NS	0.2	0.5	NS
*Pasteurellaceae_ge*	0.1	0.5	NS	4.7	5.6	NS	0.2	0.5	NS
**Neisseriaceae**	0.4	1.7	NS	3.2	5.5	NS	0.3	0.8	NS
*Conchiformibius*	0.4	1.7	NS	3.1	5.3	NS	0.3	0.7	NS
**Burkholderiaceae**	0.4	0.8	NS	1.0	2.1	NS	0.5	1.1	NS
**Gammaproteobacteria_fa**	0.7	0.8	NS	0.4	0.5	NS	1.0	1.0	NS
*Gammaproteobacteria_ge*	0.7	0.8	NS	0.4	0.5	NS	1.0	1.0	NS
**Actinobacteria**	4.7	5.2	A*	18.2	19	**B**	8.8	14.2	**AB**
**Corynebacteriaceae**	0.6	1.2	NS	4.9	7.7	NS	0.6	1.0	NS
*Corynebacterium_1*	0.2	0.4	NS	2.6	5.6	NS	0.3	0.8	NS
*Corynebacterium*	0.4	1.2	NS	1.8	5.2	NS	0.2	0.7	NS
**Micrococcaceae**	0.2	0.5	NS	6.1	16.2	NS	0.4	0.7	NS
*Rothia*	0.1	0.3	NS	6.0	16.2	NS	0.1	0.4	NS
**Microbacteriaceae**	2.9	4.7	NS	5.7	12.0	NS	6.5	8.7	NS
*Leucobacter*	2.4	4.6	NS	5.7	12.0	NS	6.4	8.7	NS
**Tenericutes**	12.8	23	NS	11.4	25.8	NS	3.7	10.8	NS
**Mollicutes_fa**	12.6	23	**A**	11.4	25.8	**AB**	3.7	10.7	B*
*Mollicutes_ge*	12.6	23	NS	11.4	25.8	NS	3.7	10.7	NS
**Firmicutes**	6.2	8.7	NS	12.3	14.5	NS	3.6	4.2	NS
**Streptococcaceae**	1.7	4.1	NS	4.8	7.1	NS	0.3	1.1	NS
*Streptococcus*	1.7	4.1	NS	4.8	7.1	NS	0.3	1.1	NS
**Staphylococcaceae**	1.3	2.8	NS	3.3	10.3	NS	1.6	3.1	NS
*Staphylococcus*	1.2	2.8	NS	3.3	10.3	NS	1.6	3.1	NS
**Aerococcaceae**	0.8	3.1	NS	1.6	3.2	NS	0.0	0.1	NS
*Abiotrophia*	0.7	3.0	NS	1.5	3.1	NS	0.0	0.1	NS
**Carnobacteriaceae**	1.1	2.6	NS	0.0	0.2	NS	0.0	0.0	NS
**Bacillales_Family_XI**	0.2	0.8	NS	1.0	1.9	NS	0.0	0.1	NS
*Gemella*	0.2	0.8	NS	1.0	1.9	NS	0.0	0.1	NS
**Clostridiales_Family_XI**	0.8	1.7	NS	0.5	0.9	NS	1.0	1.7	NS
*Helcococcus*	0.7	1.7	NS	0.4	0.8	NS	1.0	1.7	NS
**Bacteroidetes**	2.5	6.3	NS	3.3	4.3	NS	1.7	3.5	NS
**Porphyromonadaceae**	0.9	4.1	NS	1.7	3.3	NS	1.0	3.3	NS
*Porphyromonas*	0.9	4.1	NS	1.7	3.3	NS	1.0	3.3	NS
**Weeksellaceae**	1.3	3.3	NS	0.8	1.0	NS	0.5	0.9	NS
**Fusobacteria**	0.1	0.2	NS	1.0	1.8	NS	0.2	0.6	NS

Mean relative percentages (mean rel. freq) and standard deviation (SD) of the most abundant bacterial groups, annotated to the level of phylum, family and genus, based on sequencing of the V1-V3 region of the 16S rRNA gene

Group DL = mesocephalic or dolichocephalic dogs of medium to large breeds

Group B = brachycephalic dogs of small breeds

Group T = mesocephalic or dolichocephalic terrier dogs of small breeds

A,B: Operational taxonomic units not sharing a common letter between breed groups differ significantly. *p < 0.05; **p < 0.01; ***p < 0.001; ****p < 0.0001; NS not significant

The phylum Tenericutes (almost exclusively composed by an unclassified species of the class Mollicutes) was less abundant in group T compared to the two other groups. The abundance of the genus *Pseudomonas* (family Pseudomonadaceae) was increased in group T although this was mainly caused by 3 individuals (Fig. 1).

The genus *Staphylococcus,* in group B, was mainly composed of the species *Staphylococcus pseudintermedius* (3.07% (0–36.74%)). In contrast, the percentage of the species *Staphylococcus aureus* was slightly lower in group B (0.01%) compared to the two other groups (0.33% (0–4.55%) in group DL, 0.41% (0–4.97%) in group T).

**Fig. 6:**
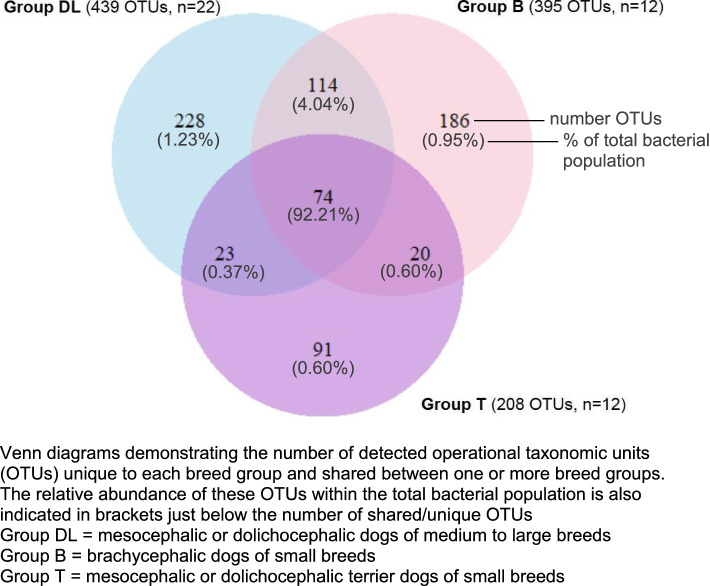
Distribution of all detected OTUs between the 3 breed groups. Venn diagrams demonstrating the number of detected operational taxonomic units (OTUs) unique to each breed group and shared between one or more breed groups. The relative abundance of these OTUs within the total bacterial population is also indicated in brackets just below the number of shared/unique OTUs. Group DL = mesocephalic or dolichocephalic dogs of medium to large breeds. Group B = brachycephalic dogs of small breeds. Group T = mesocephalic or dolichocephalic terrier dogs of small breeds

The similarities and differences in bacterial composition between the 3 groups were visualized using a Venn diagram based on the occurrences at species level (Fig. 6) and showed that 74 OTU were shared between all groups which represents 16.9, 18.7 and 35.6%, of groups DL, B, and T respectively. These 74 OTUs represent the vast majority (92.21%) of the total bacterial population. The relative proportion of unique OTUs to each group was comparable with a value around 45–50% for the 3 groups (group DL: 51.9%, group B: 47.1%, group T: 43.8%). These unique OTUs represent a small proportion (together around 3%) of the total bacterial population. As a result, the differences associated with group B were more related to differences in abundance of shared OTUs than to the presence of unique OTUs.

## Discussion

The focus of this study was to investigate variations in the NM in healthy dogs of different breeds with different facial and body conformations. To this end the NM of 46 dogs of 3 different breed groups were compared. We showed major differences in the NM composition together with increased richness and α-diversity in brachycephalic dogs, compared to meso−/dolichocephalic medium to large dogs and dogs from terrier breeds. Additionally, no specific correlations with sex or environment were determined. The influence of age remains however unclear.

In the present study, the nasal microbial population was largely dominated by the phylum Proteobacteria and the family Moraxellaceae, in accordance with what has been described in previous studies using 16S rRNA gene amplicon sequencing for bacterial analysis [7, 9, 10]. The phylum Proteobacteria has been found to dominate in association with Tenericutes and Bacteroidetes [7, 9] or in association with Firmicutes and Bacteroidetes [10] while in the current study it was associated with Tenericutes and Actinobacteria followed by Firmicutes and Bacteroidetes. The Actinobacteria phylum predominated in the group of brachycephalic dogs, likely explaining the difference with other studies in which this breed type was not sampled.

The group of brachycephalic dogs in this study was also characterized by a higher species α-diversity and richness, a decrease in Proteobacteria (mainly a decrease in *Moraxella* and *Suttonella*) associated with an increase in Actinobacteria (mainly an increase in *Corynebacterium* and *Rothia*), Firmicutes (mainly an increase in *Streptococcus* and *Staphylococcus*) and other Proteobacteria (mainly an increase in unclassified genus of the order Pseudomonadales*,* unclassified genus of the family Pasteurellaceae, and *Conchiformibius*). Different reasons could explain these changes.

A first hypothesis rests on the probable occurrence of an important reflux of oropharyngeal secretions in brachycephalic breeds [34]. Indeed, in humans, an association between severe obstructive sleep apnea, local inflammation and alterations in NM has been demonstrated [35]. Such association was presumed to be due to recurrent obstruction during sleep causing reflux of oropharyngeal secretions that otherwise would be swallowed in a healthy subject. Human oral commensals, such as *Streptococcus*, *Veillonella* and *Porphyromonas* were frequently identified in men with obstructive sleep apnea. Dogs of brachycephalic breeds frequently have at least a certain degree of upper respiratory obstruction and in the English bulldog obstructive sleep apnea has been described and even evaluated as a model for obstructive sleep apnea in humans [36]. In dogs, the oropharyngeal microbiota has been shown to be associated with higher abundance of *Porphyromonas* sp*.* (family Porphyromonadaceae), Pasteurellaceae, *Conchiformibius* sp. (family Neisseriaceae), with less *Moraxella* sp. (Moraxellaceae) and Pseudomonadaceae compared to the nasal cavities [7, 9]. Interestingly, these features corresponded to some of the variations observed in the NM of brachycephalic dogs in this study supporting the hypothesis of contamination of the NM by the oropharyngeal populations.

Another hypothesis to explain differences in NM composition in brachycephalic dogs is related to the changes in airflow distribution and increased airflow resistance described in brachycephalic breeds [32, 33]. Indeed, in one study in men investigating changes in bacterial communities after sinus surgery [37], greater airflow was suggested to cause reductions in temperature and humidity creating a cooler and drier postoperative ecosystem, with an effect on bacterial composition. According to this hypothesis, changes in intranasal temperature and humidity in brachycephalic dogs could be related to distinct intranasal microenvironment in these breeds compared to meso- and dolichocephalic dog. Unfortunately, intranasal temperature and relative humidity have not yet been analyzed in dogs.

Alternatively, the differences in NM found in brachycephalic dogs could also be associated with the reduced length of the nasal cavities in brachycephalic dogs, and the closer proximity between the nares and the deeper intranasal sites. In humans *Corynebacterium* [38], *Propionibacterium* and *Staphylococcus* genera [2] have been shown to dominate in the anterior nares compared with deeper intranasal sites. Such differences have been suspected to be secondary to niche-specific micro-environmental conditions such as pH, humidity, temperature and epithelium type [2, 6, 39], with humidity and moisture representing more favorable environmental factors for *Corynebacterium* and *Staphylococcus* species on this mucosal site [2]. In dogs, compared to the other sites of the skin, the nares were shown to be colonized by Moraxellaceae (genus *Moraxella*) in combination with Oxalobacteraceae (genus *Ralstonia*), Corynebacteriaceae (genus *Corynebacterium*) and Staphylococcaceae (genus *Staphylococcus*) [40]. Therefore, the higher predominance of *Staphylococcus* (phylum Firmicutes) and Corynebacteriaceae (phylum Actinobacteria) found in the nasal cavities of brachycephalic breeds could be associated with the closer location between the sampled site and the nares.

The presence of bacterial population of the nares in the NM of brachycephalic dogs could also be linked to contamination of the swab by the microbiota of the nares and surrounding skin, due to the reduced nasal passage in these breeds. However, such a contamination has been minimized by the use of a sterile speculum for introduction of the swab into the nasal cavity.

Finally, as only one of these hypotheses could hardly explain all the variations observed in the NM of brachycephalic dogs compared to the other breeds, most likely the differences in NM in brachycephalic dogs could be multifactorial and be initiated by more than one of these mechanisms.

As previously described [7, 9, 10] *Moraxella* was the most abundant genus in the family Moraxellaceae in this study. Despite their numerical dominance, the genus *Moraxella* and associated family Moraxellaceae were significantly lower in brachycephalic breeds compared to the 2 other breed groups. In dogs, the abundance of *Moraxella* in the nasal passages has been reported to be decreased in dogs with nasal disease (chronic rhinitis and nasal neoplasia) [10]. Whether this is a cause or consequence remains to be discovered and the exact role of *Moraxella* in the nasal cavities is still unknown. The decreased amount of *Moraxella* observed in brachycephalic dogs in this study is not in favor of this alteration being at the origin of nasal diseases, as brachycephalic dogs are less prone to these pathologies compared with the other breed groups [41]. Consequently, the decreased amount of *Moraxella* reported in dogs with nasal disease is more likely due to local changes in microenvironment secondary to the disease. This is consistent with our hypothesis that the lower abundance of Moraxellaceae in brachycephalic dogs in this study could be due to selection pressure by other taxa, such as those colonizing the nares or those brought by oropharyngeal reflux, and/or due to local changes in microenvironment as detailed earlier.

In young children, profiles dominated by *Moraxella* and *Dolosigranulum* combined with *Corynebacterium* form a stable nasal microbiome associated with lower rates of respiratory infections. With the exception of *Moraxella catarrhalis* which is described to be associated with bronchiolitis, otitis and chronic rhinosinusitis [19, 25, 42, 43], *Moraxella* might be a keystone bacterium in infants.

In dogs, the vast majority of Moraxellaceae found in this study, besides the species *Moraxella canis*, were not resolved beyond genus level. As a result, the potential presence of *M. catarrhalis* in healthy dogs is unknown and the lower abundance of *Moraxella* in brachycephalic dogs makes the hypothesis of *Moraxella* as a keystone bacterium less likely in dogs.

Patterns of microbiota sharing have been described between humans and companion animals. Humans and pets living in the same household seem to share more microbiota with each other than humans and pets living in different households [44]. Although *Staphylococcus aureus is one* of the most well-described pathobiont of the nasal cavities in men [45], its level of carriage was low in this study, particularly in brachycephalic breeds. Interestingly, an inverse correlation between the genus *Corynebacterium*, a family that was more abundant in brachycephalic breeds, and *S. aureus* has been reported in some studies of adult NM [2, 46]. However, conclusions cannot be drawn as inter-species interactions are very complex and have not been studied yet in the nasal cavities of dogs. The carriage of *Staphylococcus pseudintermedius* in companion animals has also received attention as it can also be an opportunistic pathogen [47]. In opposition to *S. aureus*, *S. pseudintermedius* was more prevalent in brachycephalic breeds. These informations could be of value when evaluating dog breeds as a potential source of Staphylococcus carriage.

Differences in NM due to age have been described in humans, but mostly between infants and adults, possibly in association with maturation of the immune system [43] and in elderly, where a nasal community shifting toward oropharyngeal population would occur [18]. In dogs, minor changes (increased Shannon diversity index of the microbiota of the nasal mucosa) have a been reported in dogs older than 9 years in one study [10], and no changes in another [9]. In the present study, age (< 3 years) appeared as being one of the variables participating to the constraint, along with breed group in the RDA analysis performed on the table of values at species level. Other analyses (RDA based on the intrinsic diversity as well as the different individual analyses) however failed to show a correlation with the age classes. Dogs of group B were significantly younger than dogs of group T (but not than dogs of group DL). As a result, the effect of age could be due to the fact that dogs of group B are younger or age could enhance the breed effect. Breed and age cannot be completely dissociated in this study cohort.

The effect of growing and ageing was not specifically addressed by including significant numbers of immature and/or very old dogs. Nevertheless, in dogs, young age has not been described as influencing the NM and beside one dog of 6 months in group B, all dogs were older than 1 year. Altogether, shifts of the NM in association with age at first sight seem to be negligible and the influence of growth or immune system immaturity on the NM of group B was unlikely to explain the differences with group T and DL.

Differences in NM between individuals living in rural and industrial locations have been reported, the latter being associated with exposure to pollution which has been hypothesized to cause microbiota alteration [15]. In dogs, one study also suggested that the NM could differ based on location [9]. Dogs of the same breed were compared between Alabama and California and differences in α- and β-diversity were observed.

Differences between dogs living in rural and industrial regions were not observed in the present study. However, in contrast to the two studies cited above, differences in geographic locations and associated environmental conditions among the dogs of this cohort were probably quite limited since all dogs were living in Belgium which is a small country with few environmental differences.

The sampling protocol described in this study aimed to limit contaminations by the nares and surrounding skin by introducing the swab through a sterile speculum and during anesthesia. Performing sampling during anesthesia allowed proper swabbing of the nasal mucosa of deeper subsites. This protocol as well as the swab that was used (Copan®, FLOQSwabs™, 553C, Brescia, Italy) seem suitable to sample the nasal microbial population as it yielded sufficient material. This is in agreement with other studies showing that swab samples are representative of the microbiome in the nasal cavities in healthy subjects [48].

Comparison across studies is made difficult by the potential introduction of bias due to variations in sampling technique, sampling site, DNA extraction, different variable regions of the 16S rRNA gene and the taxonomic database used to characterize the bacterial microbiota. It is possible that some of the differences with other publications in dogs are due to variations in sampling method. In one study the nasal mucosa was sampled in awake animals without speculum, which likely prevented sampling deep subsites and could have led to contamination by the nares and surrounding skin [10]. In another study, samples were performed in anesthetized dogs at midway between the tip of the nose and the medial canthus without speculum [7]. And finally, in yet another, swabs were sampled without anesthesia and at a distance of half an inch from the nares, so again more in the cranial part of the nasal cavities [9]. In all these studies a different region of the 16S rRNA gene was sequenced (V4 or V4-V6) and different databases were used.

Limitations in this study include the absence of information concerning anatomical spatial organization of the NM in groups DL and T as well as the absence of oro-pharyngeal and nostril swabs which would have allowed comparing the microbiota in the 3 adjacent niches, and the possible impact of reflux of oropharyngeal secretions and/or nares contamination on the NM. Statistical comparison of different types of diet was not performed due to small group size. The vast majority of them were fed with dry commercial food and although from many different brands, these diets were probably much more similar to each other, compared with the tremendous variations in diets seen in humans. The influence of other non-measured environmental parameters (season and crowding conditions for example) cannot be excluded. Even if age and living environment were not individually associated with significant changes of the NM in this study, it cannot be excluded that the combination of multiple factors (age, diet, environment) could have influenced the results. Finally, in addition to the bacterial composition, the nasal cavities host a complex viral and fungal community that has not been taken into account but could also influence the bacterial microbiota.

## Conclusion

In conclusion, healthy brachycephalic breeds and their unique facial conformation is associated with a distinct nasal microbial profile. Whether this finding is associated with the lack of predisposition for nasal diseases in brachycephalic breeds is unknown. Results of the present study contribute to the current knowledge of the composition of healthy NM in dogs which is a necessary foundation for future research aimed at identifying the impact of various perturbations, e.g., antibiotics, vaccines, infections, diseases, on the ecology of canine nasal microbial communities.

## Methods

### Study population

Client-owned healthy dogs were prospectively recruited and categorized into 3 groups including meso−/dolichocephalic dogs of medium to large breeds (group DL), brachycephalic dogs of small breeds (group B) and meso−/dolichocephalic dogs of small terrier breeds (group T). Dogs from the DL group were selected to represent a population of dogs particularly predisposed to SNA or LPR, dogs from group B were recruited as they are not predisposed to chronic nasal diseases and have a very distinct body and facial conformation. Finally, terrier dogs were selected to represent small breed dogs without the typical facial conformation of brachycephalic dogs and as being less predisposed breeds to SNA or LPR compared to dogs from the DL group.

All dogs were exempt of clinical signs and had a normal physical examination and normal hematology and biochemistry blood work. Dogs receiving antimicrobial, anti-inflammatory or immunosuppressive medication were excluded from the study. In order to investigate a possible effect of age or environmental living conditions on the NM, all dogs included were subsequently re-classified according to their age (< 3 years (age class 1), 3–8 years (age class 2) and >  8 years (age class 3)) and their environmental living conditions (living either in a city, considered as an environment with high pollution load, or in the countryside, considered as an environment with low pollution load). Owners were also asked about exposure of their pet to tobacco smoke and type of food provided.

### Sample collection

This study was approved by the ethical committee of the University of Liege (approval number: 1854) and all samples were obtained with informed and written owner consent.

**Fig. 7:**
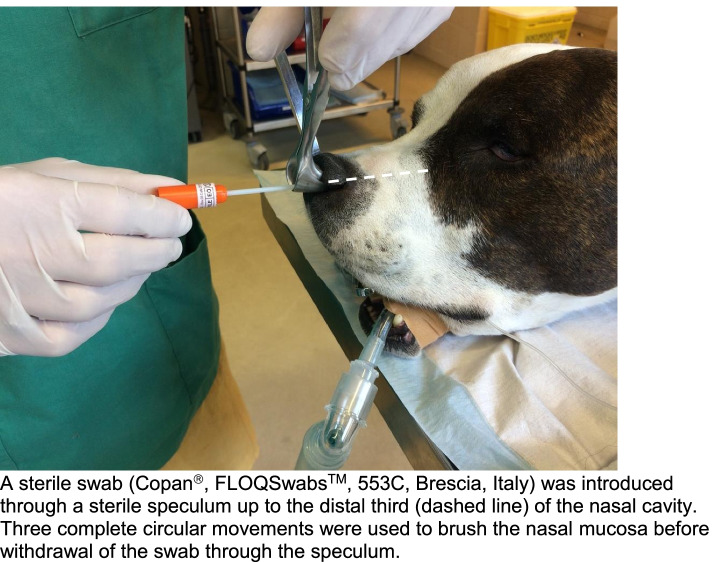
Sample collection. A sterile swab (Copan®, FLOQSwabs™, 553C, Brescia, Italy) was introduced through a sterile speculum up to the distal third (dashed line) of the nasal cavity. Three complete circular movements were used to brush the nasal mucosa before withdrawal of the swab through the speculum

Dogs were premedicated with butorphanol (0.2 mg/kg; Butomidor®, Richter Pharma) intravenously in combination with medetomidine (5 μg/kg; Medetor®, CP-Pharma). Propofol (Propovet®, Zoetis) on demand was used for induction without intubation. Under general anesthesia the nostril was maintained open using a sterile speculum, and a sterile swab (Copan®, FLOQSwabs™, 553C, Brescia, Italy) was introduced through the speculum up to the distal third of the right nasal cavity (Fig. 7). Three gentle complete circular movements were used to brush the mucosa before withdrawal of the swab through the speculum. The top of the swab was then cut, stored in a sterile cryotube and banked at − 80 °C until further analyses.

### DNA extraction and high throughput sequencing

Total bacterial DNA was extracted from all nasal swabs at the same time with the DNeasy Blood and Tissue kit (QIAGEN Benelux BV; Antwerp, Belgium) following the manufacturer’s recommendations. Spectrophotometry (NanoDrop, Thermo Scientific) was used for total DNA concentration measurement.

Bacterial 16S rRNA gene amplicons were generated via amplification of the V1-V3 hypervariable regions of the 16S rRNA gene using the following primers (with Illumina overhand adapters): forward (5′-GAGAGTTTGATYMTGGCTCAG-3′) and reverse (5′-ACCGCGGCTGCTGGCAC-3′). The DNA was purified with the Agencourt AMPure XP beads kit (Beckman Coulter; Pasadena, CA, USA) and submitted to a second polymerase chain reaction (PCR) round for indexing, using the Nextera XT index primers 1 and 2. After purification, PCR products were quantified using the Quant-IT PicoGreen (ThermoFisher Scientific, Waltham, USA). A final quantification, by quantitative PCR, of each sample in the library was performed using the KAPA SYBR” FAST qPCR Kit (KapaBiosystems; Wilmington, MA, USA) before normalization, pooling and sequencing on a MiSeq sequencer using V3 reagents (Illumina; San Diego, CA, USA). Positive control using DNA from 20 defined bacterial species and a negative control (from the PCR step) were included in the sequencing run.

Quantification of the total bacterial flora was performed with a quantitative real-time PCR targeting the V2-V3 region of the 16S rRNA gene after DNA extraction from samples. The quantitative real-time PCR were performed on the ABI 7300 real-time PCR system with Takyon™ ROX SYBR® Mastermix dTTP Blue reagents (Eurogentec, Seraing, Belgium) for a total reaction volume of 20 μl. The amplification was carried out with 40 cycles of a 95 °C denaturation phase followed by a 60 °C annealing phase. The PCR results were expressed in number of copies of the 16S rRNA gene per swab. The standard curve was based upon 10-fold dilution of a quantified PCR product. This PCR product targeting the V2-V3 region of the 16S rRNA gene was purified (Wizard® SV Gel and PCR Clean-Up System, Promega, Leiden, The Netherlands) and quantified with PicoGreen targeting double-stranded DNA (Promega) before use.

### Data analysis

Alignment and clustering were done with MOTHUR software package (v1.41.0) with an OTU clustering distance of 0.03 and based on the SILVA database (V1.32) of full-length 16S rRNA gene sequences. Vsearch [49] algorithm was used for chimera detection.

From 6,082,571 raw reads, we obtained 5,596,282 reads after cleaning (length and sequence quality). Finally, we retained 5000 reads (median 4997 reads per sample) to adjust for uneven sequencing depth across samples. Suspected contaminants were removed by filtering them from the OTU table.

### Statistical analysis

Statistical analyses were performed using XLstat (2019.3.2, Addinsoft, Paris, France), Rstudio (v1.1.463) vegan package [50] and MOTHUR (v1.40). Differences were considered significant for a *p*-value < 0.05.

The distribution of age classes was compared between the 3 breed groups (DL, B and T) using a Kruskal-Wallis test.

A redundancy analysis (RDA) on values at species level and on intrinsic diversity values (richness, evenness, alpha-diversity) were performed to evaluate the relationships between the nasal microbiota and the different potential explanatory variables (age, sex, living environment and breed group) that could influence/shape it. Forward selection was conducted to select significant variables using the “ordiR2step” function (with adjusted R^2^ coefficient) from the vegan package [50].

Individual analyses for the same variables were then carried out. Bacterial richness (Chao1 index), evenness (Simpson index-based measure), α-diversity (inverse Simpson’s index) and Good’s coverage index were obtained with MOTHUR at species level. They were compared between the 3 breed groups (DL, B and T), males and females, the 3 age classes and the 2 categories of living environment using a Kruskal-Wallis or Mann-Whitney test. Beta-diversity (bacterial community composition) at species level was assessed with MOTHUR using a dissimilarity matrix of Bray-Curtis then estimated with AMOVA (10,000 iterations) and β-dispersion was assessed with HOMOVA (10,000 iterations) in MOTHUR. NMDS plots were performed based on a Bray-Curtis dissimilarity matrix at the species level to represent the global bacterial composition (β-diversity) between groups (vegan 3D [51] and rgl [52] package). The total bacterial flora was compared between the 3 breed groups using a Kruskal-Wallis test. LEfSe was performed to detect differences in bacterial composition between breed groups at species level with MOTHUR (significant for an LDA score > 3.0 [53]). Statistical differences in the relative abundance at phylum, family, genus and species level between breed groups were assessed with a two-way ANOVA followed by a Tukey’s multiple comparison test. A Venn diagram based on the occurrences at species level was also obtained with R.

All biosample raw reads were deposited at the National Center for Biotechnology Information (NCBI) and are available under de Bioproject ID PRJNA656294.

## Data Availability

All sample raw reads associated with this study have been deposited at the National Center for Biotechnology Information (NCBI) under the accession number PRJNA656294.

## Abbreviations

NM: Nasal microbiota
LPR: Chronic lymphoplasmacytic rhinitis
SNA: Sinonasal aspergillosis
OTU: Operational taxonomic unit
AMOVA: Analysis of molecular variance
rRNA: ribosomal ribonucleic acid
NMDS: Non-metric multidimensional scaling
qPCR: Quantitative polymerase chain reaction
PCR: Polymerase chain reaction
HOMOVA: Homogeneity of molecular variance
LDA: Linear discrimination analysis
LEfSe: Linear discriminant analysis effect size
rDNA: Ribosomal deoxyribonucleic acid
B: Brachycephalic breeds
T: Terrier breeds
DL: Meso- or dolichocephalic medium to large breeds
