# Correction to: Variations in facial conformation are associated with differences in nasal microbiota in healthy dogs

**DOI:** 10.1186/s12917-022-03209-4

**Published:** 2022-03-16

**Authors:** Emilie Vangrinsven, Aline Fastrès, Bernard Taminiau, Frédéric Billen, Georges Daube, Cécile Clercx

**Affiliations:** 1 Department of Clinical Sciences, Faculty of Veterinary Medicine, University of Liege, Quartier Vallee 2, Avenue de Cureghem 3, 4000 Liege, Belgium; 2 Department of Food Sciences - Microbiology, Faculty of Veterinary Medicine, University of Liege, Quartier Vallee 2, Avenue de Cureghem 3, 4000 Liege, Belgium

**Correction to: BMC Vet Res 17, 361 (2021)**

**https://doi.org/10.1186/s12917-021-03055-w**

Following the publication of the original article [1], it was found out that the author names have been mixed up. First names and family names were interchanged. Corrected names are shown in the author group section above.

The original article has been corrected.
